# Nuclear expression of FLT1 and its ligand PGF in *FUS-DDIT3 *carrying myxoid liposarcomas suggests the existence of an intracrine signaling loop

**DOI:** 10.1186/1471-2407-10-249

**Published:** 2010-06-01

**Authors:** Mattias K Andersson, Melker Göransson, Anita Olofsson, Carola Andersson, Pierre Åman

**Affiliations:** 1 Lundberg Laboratory for Cancer Research, Department of Pathology, Sahlgrenska Academy at Göteborg University, Göteborg, Sweden

## Abstract

**Background:**

The FUS-DDIT3 fusion oncogene encodes an abnormal transcription factor that has a causative role in the development of myxoid/round-cell liposarcomas (MLS/RCLS). We have previously identified *FLT1 *(*VEGFR1*) as a candidate downstream target gene of FUS-DDIT3. The aim of this study was to investigate expression of FLT1 and its ligands in MLS cells.

**Methods:**

HT1080 human fibrosarcoma cells were transiently transfected with *FUS-DDIT3*-GFP variant constructs and FLT1 expression was measured by quantitative real-time PCR. In addition, *FLT1*, *PGF, VEGFA and VEGFB *expression was measured in MLS/RCLS cell lines, MLS/RCLS tumors and in normal adiopocytes. We analyzed nine cases of MLS/RCLS and one cell line xenografted in mice for FLT1 protein expression using immunohistochemistry. MLS/RCLS cell lines were also analyzed for FLT1 by immunofluorescence and western blot. MLS/RCLS cell lines were additionally treated with FLT1 tyrosine kinase inhibitors and assayed for alterations in proliferation rate.

**Results:**

*FLT1 *expression was dramatically increased in transfected cells stably expressing FUS-DDIT3 and present at high levels in cell lines derived from MLS. The FLT1 protein showed a strong nuclear expression in cells of MLS tissue as well as in cultured MLS cells, which was confirmed by cellular fractionation. Tissue array analysis showed a nuclear expression of the FLT1 protein also in several other tumor and normal cell types including normal adipocytes. The FLT1 ligand coding gene *PGF *was highly expressed in cultured MLS cells compared to normal adipocytes while the other ligand genes *VEGFA *and *VEGFB *were expressed to lower levels. A more heterogeneous expression pattern of these genes were observed in tumor samples. No changes in proliferation rate of MLS cells were detected at concentrations for which the kinase inhibitors have shown specific inhibition of FLT1.

**Conclusions:**

Our results imply that *FLT1 *is induced as an indirect downstream effect of FUS-DDIT3 expression in MLS. This could be a consequence of the ability of FUS-DDIT3 to hijack parts of normal adipose tissue development and reprogram primary cells to a liposarcoma-like phenotype. The findings of nuclear FLT1 protein and expression of corresponding ligands in MLS and normal tissues may have implications for tissue homeostasis and tumor development through auto- or intracrine signaling.

## Background

Myxoid/round-cell liposarcoma (MLS/RCLS) is one of the most common forms of liposarcoma and accounts for about 40% of all cases [1]. The tumor cells are characterized by the FET family [2]*FUS-DDIT3 *fusion oncogene (also called *TLS-CHOP*) present in more than 90% of cases [3-5] or the *EWS-DDIT3 *found in a minority of cases [6]. The causative role of *FUS-DDIT3 *in the initiation of MLS/RCLS and its role for the MLS-specific tumor morphology have been demonstrated in transgenic mice, xenografts and with *FUS-DDIT3 *carrying mesenchymal stem cells [7-9].

*FUS-DDIT3 *encodes a protein consisting of the N-terminal half of the FUS protein juxtaposed to the DNA-binding basic leucine zipper transcription factor DDIT3 (also known as CHOP or GADD153) [4,5]. The FUS-DDIT3 protein acts as an abnormal transcription factor [10] and the development of myxoid liposarcomas is thus regarded as a consequence of deregulated FUS-DDIT3 target genes [8,9,11]. In this study, we have investigated the expression of the putative FUS-DDIT3 target gene *FLT1 *and its encoded receptor tyrosine kinase in MLS cells.

## Methods

### Cell lines

The myxoid liposarcoma cell lines MLS 402-91, MLS 1765-92, MLS 2645-94 [3,11] and human fibrosarcoma cell line HT1080 were kept frozen in liquid nitrogen or cultured at 37°C and 5% CO_2 _in RPMI 1640 medium with HEPES buffer supplemented with 2 mM L-glutamine, 50 U/ml penicillin, 50 μg/ml streptomycin and 10% fetal bovine serum (Invitrogen). Cell lines HT1080 FUSA-GFP, HT1080 DDIT3-GFP and HT1080 FUS-DDIT3-GFP were generated by plasmid transfection of HT1080 fibrosarcoma cells as described elsewhere [8]. G418 (200 μg/ml) was constantly added to cell lines HT1080 FUSA-GFP, HT1080 DDIT3-GFP and HT1080 FUS-DDIT3-GFP to ensure stable expression of GFP constructs in the cell population. In a growth inhibition assay, FLT1-blocking antibody AF 321 (R&D systems) was added to MLS cells precultured in 4% fetal bovine serum for 14 hours in 96 well plates as described [12]. The cultures were visually analyzed by light microscopy after 72 hours of incubation. Flt-1 siRNA (sc-29319), PGF siRNA (sc-44027) and control siRNA-A (sc-37007) were transfected into cells using the siRNA Transfection Reagent (sc-29528, Santa Cruz Biotechnology) according to instructions supplied by the manufacturer.

### Quantitative real-time PCR analysis

Total RNA was prepared with the RNeasy lipid tissue kit (Qiagen) from an abdominal subcutaneous adipose tissue biopsies of healthy individuals and from isolated adipocytes as previously described [13]. Acid guanidinium thiocyanate-phenol-chloroform extraction was used to isolate total RNA in representative tumor tissue from patients diagnosed with myxoid liposarcoma. Total RNA of cultured cells was isolated using QIAshredder and RNeasy Mini Kit (Qiagen). RNA concentrations were measured with the NanoDrop ND-1000 spectrophotometer. cDNA was generated using a QuantiTect Reverse transcription kit (Qiagen) or alternatively using oligo dT primers and Superscript III reverse transcriptase (Invitrogen). Real-time PCR was performed using a 7500 Fast real-time PCR system (Applied Biosystems) with SYBR Green detection (Qiagen). Formation of expected PCR products were confirmed by agarose gel electrophoresis and melt curve analysis. Gene expression data was normalized against *ACTB *and *GAPDH *expression by geometric averaging [14]. Independent samples t-test was used to compare *FLT1 *expression between stably transfected cells. The following primer pairs were used for quantitative real-time PCR (5'-3'): FLT1_F TCCCTTATGATGCCAGCAAGT, FLT1_R CCAAAAGCCCCTCTTCCAA; PGF_F GTTCAGCCCATCCTGTGTCT, PGF_R AACGTGCTGAGAGAACGTCA; VEGFA_F CATCCTGTGTGCCCCTGA, VEGFA_R TTGTCTTGCTCTATCTTTCTTTGG; VEGFB_F AGTGCTGTGAAGCCAGACA, VEGFB_R GGAGTGGGATGGGTGATG; ACTB_F GCCGTCTTCCCCTCCATC, ACTB_R GCCTCGTCGCCCACATAG; GAPDH_F TCAGCCGCATCTTCTTTTG, GAPDH_R GACTCCGACCTTCACCTTC.

### Immunohistochemistry and immunofluorescence

Formalin-fixed paraffin embedded tissue samples were obtained, prepared and analyzed according to routine pathology protocols as part of clinical diagnostics at the Sahlgrenska University Hospital, Gothenburg, Sweden. Sections were mounted on glass slides, deparaffinated in xylol and stained as previously described [15] with primary antibodies for FLT1 (C-17, sc-316, Santa Cruz Biotechnology) and VEGF receptor 1 (ab2350, Abcam). Human normal organs and various cancers tissue arrays (Super Bio Chips) containing 59 and 60 core biopsies per slide were stained as above. Angiosarcoma tissue sections were used as positive controls for the FLT1 staining. Negative controls were performed by omitting primary antibodies. For immunofluorescence studies, cells were grown in flaskettes, rinsed in phosphate buffered saline (PBS) and fixed for 20 minutes in 4% formaldehyde in PBS. Fixed cells were stained with antiserum for FLT1 (C-17, sc-316, Santa Cruz Biotechnology) and PGF (ab9542, Abcam) and visualized using goat anti-rabbit Cy3-conjugated secondary antibodies (Dako). Slides were mounted using Prolong Gold antifade with DAPI (Molecular Probes) and allowed to cure overnight. Cellular fluorescence was imaged using a Zeiss LSM510 META confocal microscope system.

### Western blot

Cellular nuclear and cytoplasmic fractions were isolated using a PARIS kit (Ambion) according to the instructions by the manufacturer. Protein concentrations in fractions were determined using a bicinchoninic acid (BCA) protein assay kit (Pierce) and diluted for equal loading on gels. Samples were mixed with 4X LDS sample buffer (Invitrogen), 10% 0.5 M dithiothreitol and run on NuPage 4-12% Bis-Tris gels (Invitrogen). Proteins were blotted onto PVDF membranes (Immobilon) and probed with antibodies for FLT1 (C-17, sc-316, Santa Cruz Biotechnology), Lamin A (133A2, ab8980, Abcam) and GAPDH (mAbcam 9484, Abcam). Bands were visualized by horseradish peroxidase-conjugated secondary antibodies by chemiluminscent detection (SuperSignal West Dura Extended Duration Substrate, Pierce). Chemiluminscent membranes were imaged using a LAS-4000 imaging system (Fujifilm).

### Proliferation assay

Tumor cells were seeded out in wells of black walled/clear bottom 96-well plates (BD Falcon) and allowed to adhere overnight. The following day, VEGFR tyrosine kinase inhibitors (TKI) II or III (Calbiochem) were added in a concentration range of 0 to 10 μM. After 72 hours, cells were subjected to multiple freeze-thaw lyses at -80°C and 37°C, respectively, where after cell proliferation relative to untreated control cells was assayed with a CyQuant Cell Proliferation Assay Kit (Molecular Probes) using a Wallac 1420 multilabel counter (PerkinElmer). Independent samples t-test was used to evaluate differences between wells.

## Results and Discussion

**Figure 1:**
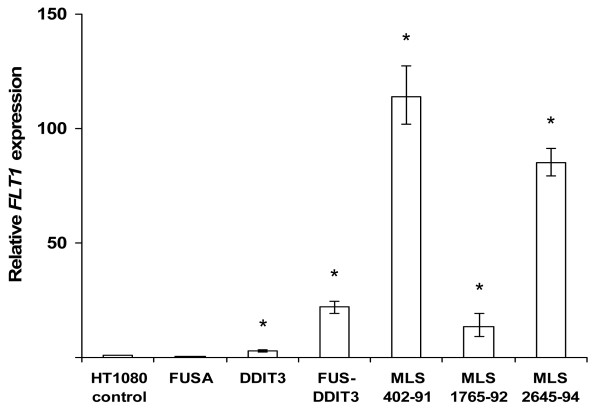
**Increased *FLT1 *transcription in FUS-DDIT3 expressing cell lines**. Bars show mean relative *FLT1 *expression by quantitative real-time PCR analysis of three independent biological replicates compared to wild type HT1080 with *FLT1 *expression set to 1. The geometric mean of *ACTB *and *GAPDH *expression was used to normalize *FLT1 *expression between samples. Error bars show standard error of the mean. Asterisks indicate statistical significance with p < 0.01.

Inspection of previously collected microarray data [8] identified *FLT1 *as a candidate downstream target gene for FUS-DDIT3. *FLT1 *encodes the Fms-like tyrosine kinase 1 (also called vascular endothelial growth factor receptor 1, VEGFR1) reported to be involved in autocrine growth stimulatory loops in other sarcoma types [12]. Here we report that HT1080 human fibrosarcoma cells stably transfected with *FUS-DDIT3 *express *FLT1 *transcripts more than 20 times stronger than the original HT1080 cells (Figure 1). In addition, *DDIT3*-transfected HT1080 cells showed minor but significant (< 3 times) increase in *FLT1 *expression whereas HT1080 cells transfected with the 5' part of *FUS *showed slightly downregulated *FLT1 *expression. Quantitative PCR (QPCR) analysis also revealed that three tested MLS-derived cell lines expressed high levels of *FLT1 *transcripts, further indicating that *FLT1 *is expressed in *FUS-DDIT3 *carrying cells (Figure 1). However, transient transfection of HT1080 cells with *FUS-DDIT3 *failed to induce an increased *FLT1 *expression during a 48 hour observation period (Additional file 1). This indicates that transcription of *FLT1 *is not a direct target of FUS-DDIT3. Instead, the change in *FLT1 *expression could be an indirect effect of FUS-DDIT3 expression. The FUS-DDIT3 protein has the capacity to reprogram HT1080 cells to a liposarcoma-like phenotype by hijacking parts of normal adipose tissue development [8,16]. The induction of *FLT1 *transcription in FUS-DDIT3 expressing cells may thus be a part of this process. This explanation is further supported by QPCR analyses showing prominent expression of *FLT1 *transcripts in isolated normal adipocytes (Additional file 2).

**Figure 2:**
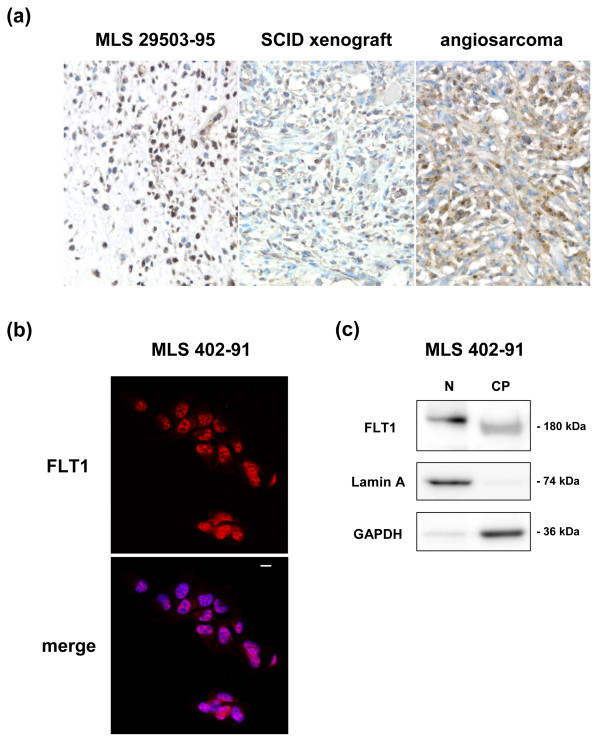
**Nuclear FLT1 localization in MLS tumors and cultured MLS cells**. **(a) **Immunohistochemical analysis of FLT1 expression in tissue sections of a representative MLS tumor and in an MLS 402-91 SCID mouse xenograft. Brown staining indicates FLT1 expression while blue staining shows negatively staining nuclei. Cytoplasmic FLT1 expression in angiosarcoma was used as a positive control. **(b) **FLT1 expression in cultured MLS 402-91 cells analyzed by immunofluorescence. The merge image shows nuclear DAPI staining in blue. Scale bar indicates 10 μm. **(c) **Western blot analysis of nuclear (N) and cytoplasmic (CP) fractions of MLS 402-91 cells. Lamin A was used as a control for the nuclear fraction and GAPDH was used as control for the cytoplasmic fraction.

Immunohistochemistry (IHC) analysis of human MLS tissues from nine cases and an MLS cell line xenografted in SCID mice showed strong, predominantly nuclear expression of the FLT1 protein (Figure 2a). The FLT1 nuclear stained tumor cells ranged from 60-80% in the nine investigated cases. No correlation between FLT1 staining and diagnosis (MLS or RCLS) were found. Immunofluorescence analysis of *in vitro *cultured MLS-derived cell lines 402-91 (Figure 2b) and 2645-94 (not shown), also showed a nuclear FLT1 localization. IHC staining of angiosarcoma tissue showed a cytoplasmic FLT1 staining in agreement with earlier reports [17] (Figure 2a). Indistinguishable results were obtained with two different antibodies for FLT1.

The nuclear localization of FLT1 in MLS/RCLS was surprising since the protein is commonly reported a cytoplasmic or plasma membrane bound protein [18]. To further probe the cellular distribution of the FLT1 protein, we prepared nuclear and cytoplasmic extracts from the MLS-derived cell line MLS 402-91. Western blot analysis of these extracts with an FLT1 specific antibody showed that anti-FLT1 reactive proteins were enriched in the nuclear fraction (Figure 2c). The western blot results thus confirmed the nuclear localization of FLT1 protein in MLS cells. The migration difference detected between nuclear and cytoplasmic FLT1 (Figure 2c) could be due to different ionic strengths of the extraction buffers used for fractionation but may well be a result of post-translational modifications of nuclear FLT1 proteins.

**Figure 3:**
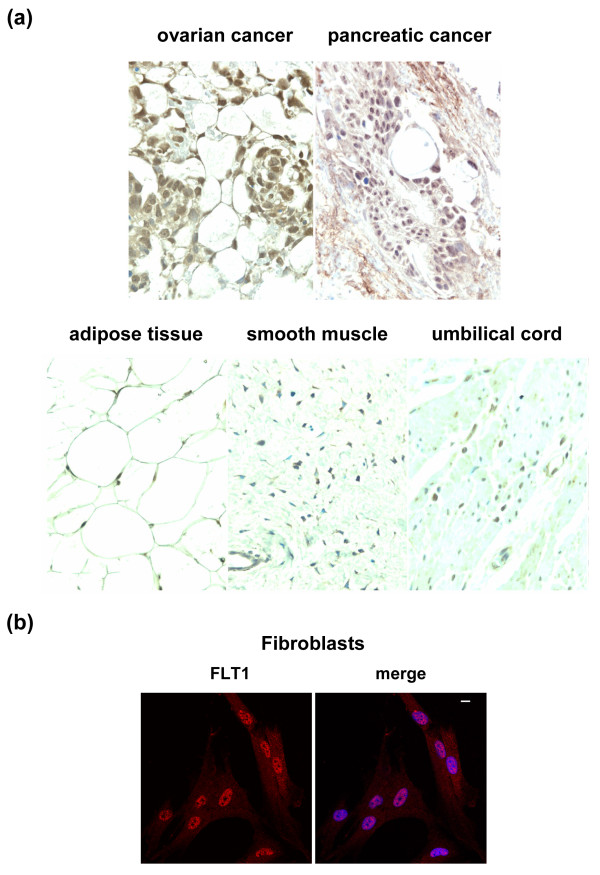
**Nuclear FLT1 expression in malignant and normal cells**. **(a) **Immunohistochemical analysis of FLT1 expression in cancerous and normal tissues. Brown staining indicates FLT1 expression while blue staining shows negatively staining nuclei. **(b) **Immunofluorescence analysis of FLT1 expression in cultured human fibroblasts. The merge image shows nuclear DAPI staining in blue. Scale bar indicates 10 μm.

These results prompted a further investigation as to whether FLT1 has a nuclear localization in other tumor types and in normal tissues. IHC analysis of tissue arrays containing 59 normal and 60 tumor-derived tissues showed a strong nuclear FLT1 expression in more than 90% of the tumor cells in 1/1 pancreatic carcinoma and in 1/3 ovary carcinomas (Figure 3a). Nuclear staining was also observed in several normal cell types, indicating that this localization of FLT1 is not restricted to tumor cells (Figure 3a). Most notably, FLT1 antibodies stained the nuclei of adipocytes, suggesting that nuclear FLT1 expression is a normal feature of this cell type. In addition, FLT1 antibodies stained the nuclei of normal skin-derived cultured F470 fibroblasts but with a more prominent cytoplasmic staining compared to the MLS cells (Figure 3b). The observation that FLT1 has a nuclear localization in some tissues may indicate additional, yet unknown, functions for this protein. Nuclear expression of FLT1 has also been reported in endothelial cells and in these cells FLT1 activation was necessary for growth and survival [19]. Nuclear localization of other receptor tyrosine kinases and specific functions in this compartment has previously been reported [20-23]. In MLS/RCLS and in normal adipocytes the nuclear expression may be important for maintenance of cell lineage/differentiation through phosphorylation/activation of specific chromatin components or transcription factors.

**Figure 4:**
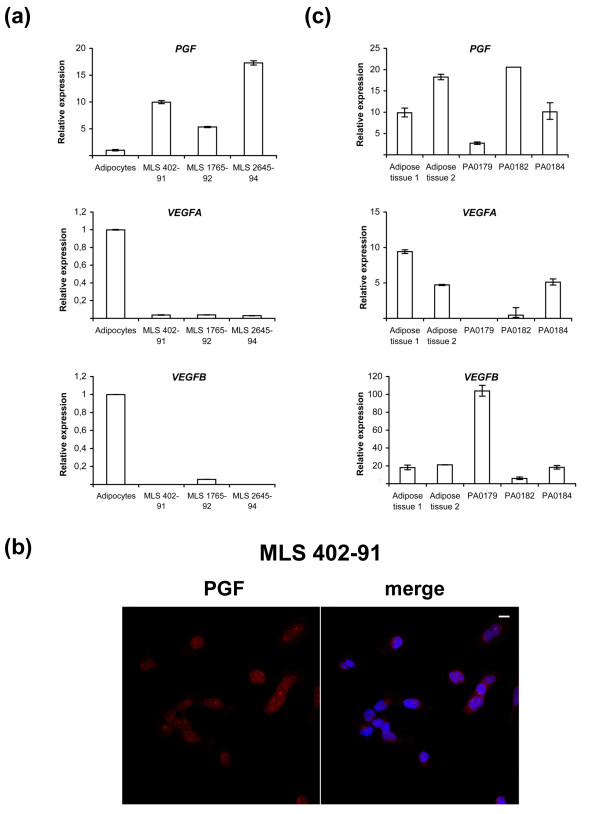
**Quantitative real-time PCR analysis of *PGF*, *VEGFA *and *VEGFB *expression in FUS-DDIT3 expressing cell lines and tumors**. **(a) **Bars show fold difference in ligand expression compared to adipocytes with a relative expression set to 1. Error bars show standard error of the mean **(b) **Immunofluorescence analysis of PGF expression in MLS 402-91. The merge image shows nuclear DAPI staining in blue. Scale bar indicates 10 μm. **(c) **Bars show relative expression of ligand mRNA in normal adipose tissue and in three MLS tumors. The geometric mean of *ACTB *and *GAPDH *expression was used to normalize gene expression between samples. Error bars show standard error of the mean.

**Figure 5:**
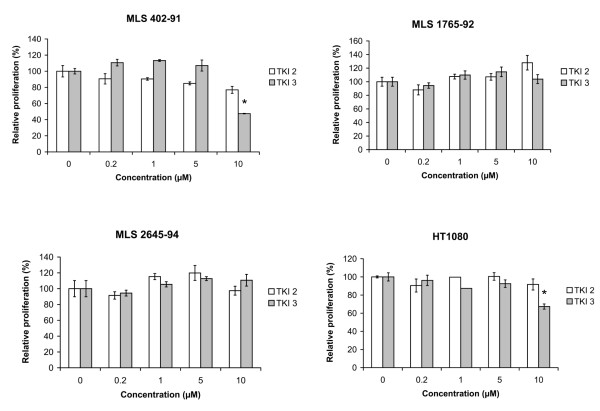
**Proliferation of tumor cells treated with VEGFR tyrosine kinase inhibitors**. Cells were treated with indicated concentrations of drugs for 72 hours and relative cell proliferation was assayed in comparison to untreated control cells. Bars show means of three biological replicates and error bars show standard error of the mean. Asterisks indicate significant inhibition of proliferation with p < 0.01.

Further inspection of array data suggested that *PGF*, encoding placental growth factor (PGF), a ligand of FLT1, is transcribed in MLS cells. Quantitative RT-PCR analysis of MLS-derived cell lines showed that *PGF *expression was elevated in these cells compared with normal adipocytes (Figure 4a). The other FLT1 ligands *VEGFA *and *VEGFB *were expressed in cultured MLS cells but to a much lower degree than in adipocytes. In addition, the MLS cell lines showed PGF expression detected by immunofluorescence analysis (Figure 4b). In contrast to MLS cell lines, three MLS tumors showed a much more heterogenous expression of *PGF*, *VEGFA *and *VEGFB *but all tumors expressed *PGF *to some degree (Figure 4c). These results suggest differences of *in vivo *and *in vitro *ligand expression. Still, in light of our results, we hypothesize that autocrine circuits are operating in MLS cells. Such autocrine loops involving FLT1 have been reported to contribute to growth and survival of tumor cells [12,24,25] and to regulate differentiation of normal cell types [26,27]. We tested for the presence of a possible FLT1 autocrine growth stimulatory loop in MLS cell line 402-91 by using an FLT1-blocking antibody reported to inhibit the growth of cells that are dependent on FLT1 signaling [12]. Incubation with this antibody failed however to affect growth or survival of the MLS cells. Though, receptor-targeted antibodies may be ineffective if the receptor and ligand interacts inside of the plasma membrane, which could be the case here. Such intracrine growth stimulatory circuits were recently reported for FLT1 in mammary carcinoma cells [28]. To test for an intracrine circuit in MLS/RCLS cells, knockdown experiments using siRNAs against *FLT1 *and *PGF *mRNA were attempted. As previously reported for knockdown experiments in MLS/RCLS cells [29], we were not able to achieve efficient knockdown of *FLT1 *or its ligand gene *PGF*. Instead we employed two specific inhibitors of FLT1 kinase activity (TKIs) in proliferation assays of MLS cell lines. None of the kinase inhibitors caused any decrease in cell numbers within the concentration range where they have shown specific inhibition of FLT1 activity [30,31] (Figure 5). Thus, it is not likely that FLT1 and a probable autocrine/intracrine loop has a role in survival or proliferation in MLS cells but may contribute to the liposarcoma phenotype. Treatment with the TKIs gave no effect on the expression or localization of FLT1 in the MLS cells. Thus, the nuclear localization appears not to be related to the phosphorylation status of FLT1.

## Conclusions

We conclude that upregulation of *FLT1 *in MLS/RCLS is an indirect downstream effect of the liposarcoma fusion oncogene *FUS-DDIT3 *and that FLT1 is expressed as a nuclear protein both in MLS/RCLS lipoblasts and in normal adipocytes. The nuclear expression of FLT1 in adipocytes and other normal cell types suggest that this receptor tyrosine kinase has cell type specific functions in the nuclear compartment. Most likely these involve maintenance of cell lineage through phosphorylation of target proteins residing in the nucleus. The expression of FLT1 ligand genes *PGF*, *VEGFA *and *VEGFB *in MLS/RCLS cells and in normal adipocytes further indicates the presence of possible autocrine or intracrine loops. The importance of FLT1, PGF, VEGFA and VEGFB expression and their functions in MLS/RCLS tumor development remain unclear but our results point out this receptor/ligand system as operational in MLS/RCLS and as such a potential target for intervention in this tumor type.

## Abbreviations

CHOP: CCAAT/enhancer-binding protein homologous protein; DDIT3: DNA-damage-inducible transcript 3; FLT1: Fms-like tyrosine kinase 1; FUS: fusion (involved in t(12;16) in malignant liposarcoma); GADD153: Growth arrest and DNA-damage-inducible protein 153; GFP: green fluorescent protein; IHC: immunohistochemistry; MLS/RCLS: myxoid/round-cell liposarcomas; PGF: placental growth factor; QPCR: quantitative polymerase chain reaction; siRNA: short interfering RNA; TKI: tyrosine kinase inhibitor; TLS: translocated in liposarcoma; VEGFR1: vascular endothelial growth factor receptor 1

## Competing interests

The authors declare that they have no competing interests.

## Authors' contributions

MG and PÅ conceived of the study. MKA, MG and PÅ designed the experiments. MKA, MG, AO, CA and PÅ performed research. MKA and PÅ wrote the manuscript. All authors read and approved the final version of the manuscript.

## Pre-publication history

The pre-publication history for this paper can be accessed here:

http://www.biomedcentral.com/1471-2407/10/249/prepub

## Supplementary Material

Additional file 1 ***FLT1 *expression in transiently transfected cells**. HT1080 cells were transiently transfected with a FUS-DDIT3 construct and FLT1 expression was analyzed by quantitative real-time PCR at time points indicated in the figure. *GAPDH *expression was used to normalize *FLT1 *expression. Error bars show standard error of the mean. Click here for file

Additional file 2 ***FLT1 *expression in isolated normal adipocytes**. Expression of *FLT1 *in HT1080 cells and isolated adipocytes by quantitative real-time PCR. The geometric mean of *ACTB *and *GAPDH *expression was used to normalize gene expression between samples. Error bars show standard error of the mean. Click here for file
