# Supplementary figures and images for: Nuclear expression of FLT1 and its ligand PGF in FUS-DDIT3 carrying myxoid liposarcomas suggests the existence of an intracrine signaling loop
Source: BMC Cancer. 2010 Jun 1;10:249. doi: 10.1186/1471-2407-10-249 (PMC2889895; doi:10.1186/1471-2407-10-249)

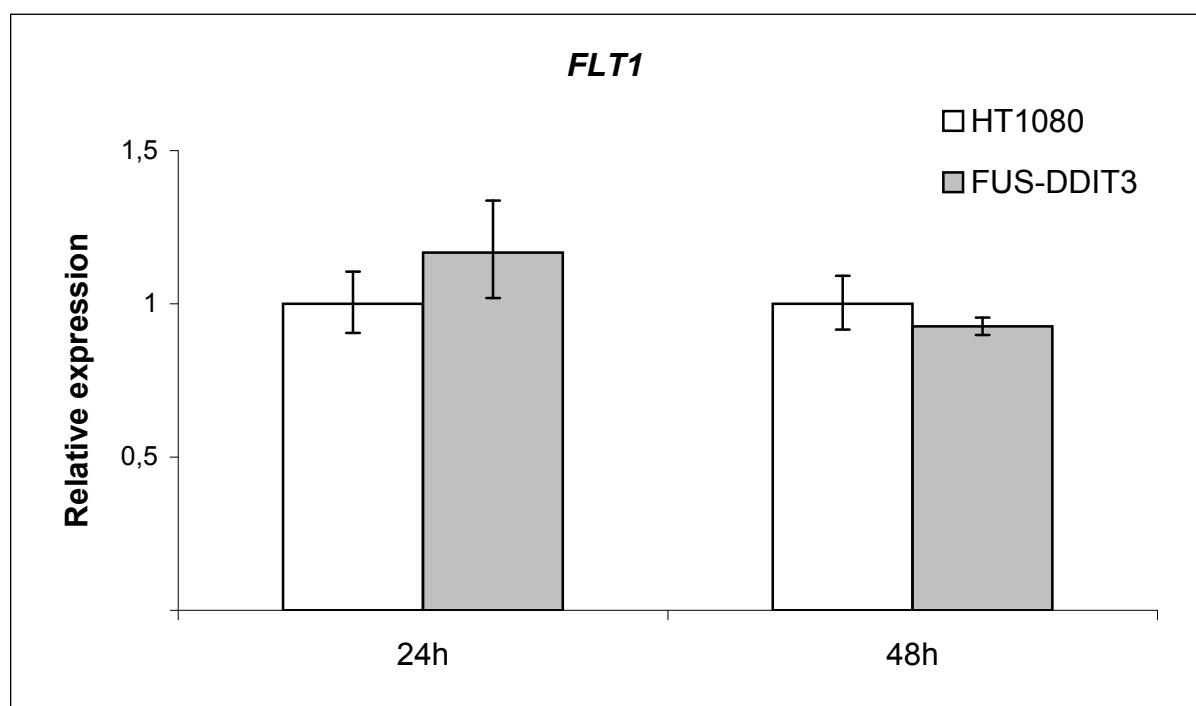

Supplement: Additional file 1 — FLT1 expression in transiently transfected cells. HT1080 cells were transiently transfected with a FUS-DDIT3 construct and FLT1 expression was analyzed by quantitative real-time PCR at time points indicated in the figure. GAPDH expression was used to normalize FLT1 expression. Error bars show standard error of the mean. [file 1471-2407-10-249-S1.PDF]

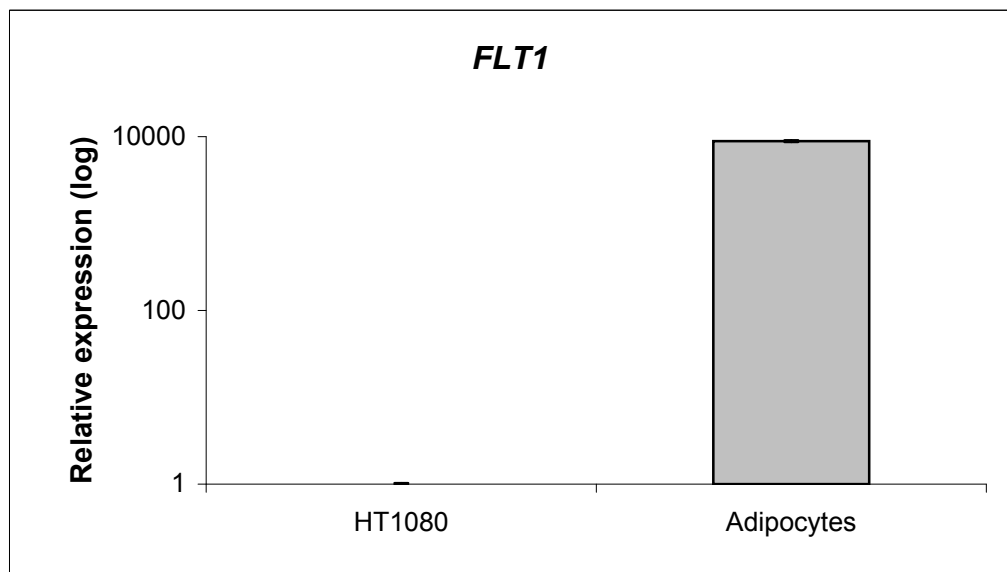

Supplement: Additional file 2 — FLT1 expression in isolated normal adipocytes. Expression of FLT1 in HT1080 cells and isolated adipocytes by quantitative real-time PCR. The geometric mean of ACTB and GAPDH expression was used to normalize gene expression between samples. Error bars show standard error of the mean. [file 1471-2407-10-249-S2.PDF]
